# Tension pneumoperitoneum caused by rupture of intraabdominal soft tissue emphysema in a child supported with high-frequency oscillatory ventilation: a case report

**DOI:** 10.1186/s13256-019-2224-3

**Published:** 2019-08-26

**Authors:** Pussayaban Suwankeeree, Sudarat Jungkraisri, Paiboon Sookpotarom, Paisarn Vejchapipat

**Affiliations:** 1 Department of Pediatrics, Panyananthaphikkhu Chonprathan Medical Center, Srinakharinwirot University, Nonthaburi, 11120 Thailand; 2 Department of Surgery, Panyananthaphikkhu Chonprathan Medical Center, Srinakharinwirot University, 222 Tiwanon Road, Pak Kret, Nonthaburi, 11120 Thailand; 3 Department of Surgery, Faculty of Medicine, Chulalongkorn University, Bangkok, 10330 Thailand

**Keywords:** Case report, Pneumoperitoneum, Emphysema, Pneumothorax, High-frequency oscillatory ventilation

## Abstract

**Background:**

We reported a case with tension pneumoperitoneum while being on high-frequency oscillatory ventilation.

**Case presentation:**

A 12-month-old Thai girl presented with acute respiratory distress syndrome, septic shock, and bacterial pneumonia. Although supported with mechanical ventilation, she still had severe hypoxia. She was then transitioned to high-frequency oscillatory ventilation. During a weaning period on day 7, she developed left tension pneumothorax requiring intercostal drainage and a markedly large amount of pneumoperitoneum. In spite of a bedside abdominocentesis, her abdomen was still tense and her hemodynamics was unstable. Subsequently, to exclude hollow viscus perforation, diaphragmatic injury caused by intercostal drainage, or abdominal compartment syndrome, she was transferred for surgery. There was no intestinal perforation. Postoperatively, she was on oxygen therapy, on chest physical therapy, and kept hemodynamically stable until she had recovered.

**Conclusion:**

A case of tension pneumoperitoneum probably caused by high-frequency oscillatory ventilation was reported. Awareness of this condition should be included in the differential diagnosis.

## Introduction

Pneumoperitoneum is generally caused by alimentary tract injury; however, pneumoperitoneum occasionally occurs as a rare presentation of barotrauma caused by ventilatory support. Most of the time, ventilator-related pneumoperitoneum can be safely treated by non-surgical means [1–6]. However, this therapeutic practice might not be implemented in all cases. We report a case with tension pneumoperitoneum while being on high-frequency oscillatory ventilation (HFOV).

### Patient information

A female patient had a history of prematurity and primary abdominal repair for gastroschisis at birth. There was no genetic investigation with respect to the congenital anomalies. There were no other associated anomalies. Subsequently, she was adopted, reared, and cared for by caregivers at a children’s center. The presentation of this reported case was the first episode of significant sickness in her life.

### Timeline

#### Prenatal history

There was a lack of prenatal history.

#### Past illness

She suffered from premature delivery with unknown actual gestation and gastroschisis at birth. She underwent primary abdominal closure and recovered uneventfully.

#### Family and psychosocial history

Because of the inability of the parents to rear their child, she was adopted to live in a children’s center. She grew up well and this presentation was her first severe illness after the birth defects. There was no record of any interventions for any diseases in the past in her individual recorded file.

#### Past history

There was no medical record of any severe condition except some minor reports of upper respiratory tract infection.

#### Presenting concerns

She presented with signs and symptoms of acute respiratory distress syndrome (ARDS). Her clinical and laboratory findings which were consistent with bacterial pneumonia were construed as a primary cause of her severe illness. Consequently, she was supported with conventional ventilation and then switched to HFOV. After approximately a week of this support, unexpected events, that is, tension pneumothorax, pneumomediastinum, and pneumoperitoneum, occurred during a step of weaning.

#### Diagnosis

The diagnosis of primary pneumoperitoneum was made. However, following an abdominocentesis, she was still hemodynamically unstable.

#### Intervention

She was planned to be treated conservatively but she was then transferred to an operating room to exclude some surgical causes.

#### Outcome

After 20 days following surgery, she improved with oxygen therapy and chest physical therapy and uneventfully recovered. She also did well at 2-week follow-up.

## Case presentation

A 12-month-old Thai girl was brought to our emergency department with high-grade fever, cough, and dyspnea. Her underlying disease was gastroschisis. She underwent primary abdominal closure at birth without complications.

Initial findings showed hypoxia, suprasternal and subcostal retraction, and crepitation on chest auscultation. According to these signs and symptoms, an initial provisional diagnosis of ARDS and septic shock had been made. All later investigations led to a diagnosis of severe bacterial pneumonia as a primary cause of ARDS. Upon examination, she had a body temperature of 39.0 ºC with a heart rate of 170 beat per minute, a respiratory rate of 70 breaths per minute, an arterial blood pressure of 70/30 mmHg, and an oxygen saturation of 80%. She looked drowsy with dyspnea. Mild cyanosis was seen on her lips. In addition to the abnormal sound found with stethoscope, we noticed that there was an abdominal paradoxical respiration. With respect to the conditions, she was intubated and supported with conventional mechanical ventilation (CMV) at a mode of pressure control with positive end-expiratory pressure of 12 mm H_2_O and total inspiratory pressure of 30 mmHg. Despite the aggressive management, she still had severe hypoxia with a partial pressure of oxygen (PaO_2_) of 82 mmHg. Consequently, she was transitioned to HFOV after 6 hours of CMV.

**Fig. 1:**
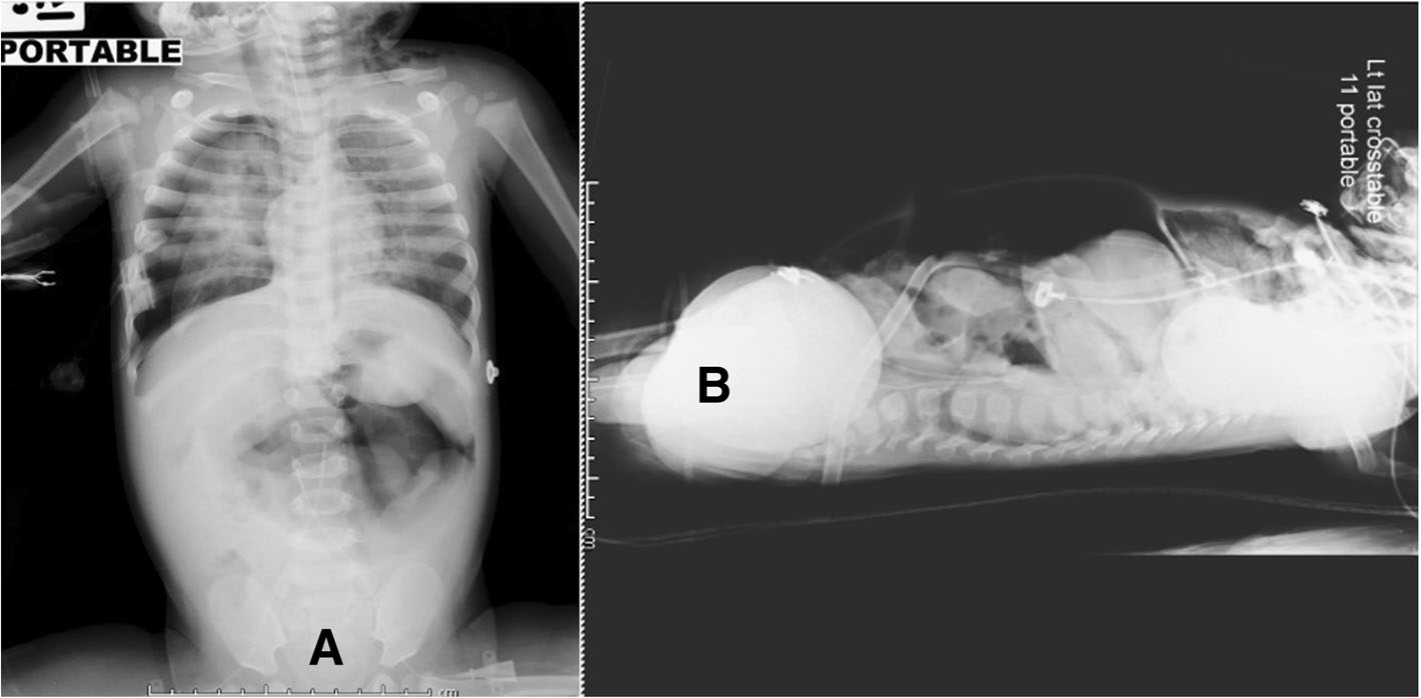
A plain thoracoabdominal film showed pneumothorax, pneumomediastinum, and a tension pneumoperitoneum in anteroposterior (**a**) and lateral (**b**) view

During the first 2 days of support with the maximum amplitude of HFOV and mean airway pressure of 32 mmHg, she was able to be maintained with an oxygen saturation of over 88%. Her condition seemed to uneventfully improve. During a weaning of mean airway pressure on day 7 of HFOV support, however, she developed an abrupt onset of inadequate ventilation and oxygenation. A radiography revealed left tension pneumothorax requiring intercostal drainage (ICD). A few hours after ICD, she suffered from a markedly distended abdomen with subcutaneous emphysema across her entire upper torso. Again, plain thoracoabdominal film showed pneumothorax with pneumomediastinum and a markedly large amount of pneumoperitoneum (Fig. 1). Her abdominal circumference suddenly increased from 40 to 50 cm. She underwent a bedside abdominocentesis withdrawing approximately 1 L of air to release intraabdominal pressure, partially improving her hemodynamic instability. Her abdomen was still tense and her hemodynamics was unstable. Subsequently, to exclude diaphragmatic injuries secondary to ICD, hollow viscus perforation, or abdominal compartment syndrome as a primary cause, she was transferred for surgery. As she had financial support from the government, there was no financial challenge for her further management.

**Fig. 2:**
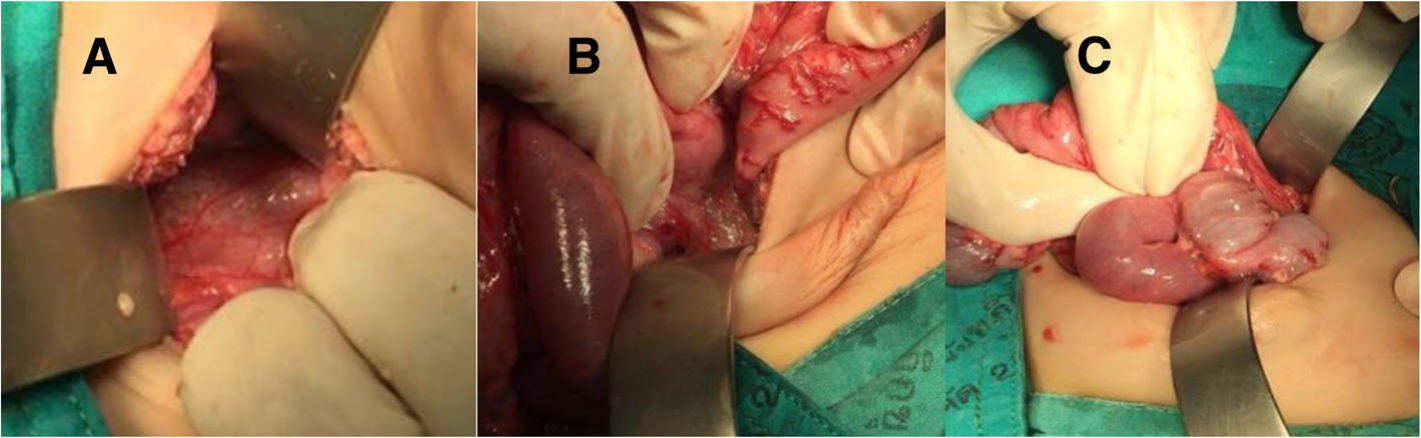
Soft tissue emphysema was found in several places, including esophagogastric junction (**a**), mesenteric root (**b**), and terminal ileal serosa (**c**)

With an upper transverse incision, soft tissue emphysema was found in several places, including esophagogastric junction, mesenteric root, and terminal ileal serosa (Fig. 2). There was no fluid or blood intraabdominally. There was no intestinal perforation and the lesions were left in place. There were no lesions on both sides of her diaphragm. Therefore, the presence of intraabdominal air was certainly due to emphysematous leakage at the aforementioned sites not a passage through diaphragm or intestinal perforation. There was no complication in the postoperative period. After 20 days in an intensive care unit, she was extubated and transferred to a pediatric ward. She was on oxygen therapy, on chest physical therapy, and kept hemodynamically stable until she had recovered. She was doing well at the time of 2-week interval follow-up. Currently, 12 months later, she is well without any sequelae from surgery.

## Discussion

Pneumoperitoneum usually constitutes a surgical condition requiring intraabdominal repair of alimentary tract injuries. However, many cases of patients with non-surgical pneumoperitoneum have been reported in the literature [1–6]. The condition affects patients of all ages and both genders. In the pediatric age group, neonates and infants were the most affected and almost all patients were involved with CMV. Barotrauma, generated by this type of ventilation, would be a cause of alveolar rupture. However, this generally results in pneumothorax, subcutaneous or interstitial emphysema, and pneumomediastinum [7–11]. Pneumoperitoneum related to mechanical ventilation is an unusual condition. The plausible mechanism would be increased pressure that causes bidirectional airflow, leaking along perivascular sheaths to the hilum and, rarely in some cases with pleuroperitoneal anatomical defects, dissecting to retroperitoneum [5, 11, 12].

The patient described here was unusual in that severe barotrauma did not develop in a phase of support with CMV but occurred in the weaning phase of HFOV. HFOV is known to reduce air leakage in patients with poor lung compliance, particularly with pathology like ARDS. HFOV is considered a lung-protective strategy since it generates smaller tidal volume with high frequency and provides adequate ventilation with a relatively smaller pressure than CMV. Our patient developed pneumothorax and pneumoperitoneum on the seventh day of HFOV at mean airway pressure of 24 mmHg which was not a maximal mean airway pressure (32 mmHg). In general, during HFOV support in patients with very low lung compliance, mean airway pressure underestimates mean alveolar pressure and will reflect the alveolar pressure when their condition of lung parenchyma improves [13–15]. Hence, our patient was affected with pneumoperitoneum at a low pressure of mean alveolar pressure.

There is a dilemma in the treatment of patients with spontaneous pneumoperitoneum. Many reported cases could be safely treated with non-surgical treatment by an awareness of this condition [7, 16, 17]. Our patient possessed some risks factors of non-surgical pneumoperitoneum, including infant period, ARDS with ventilatory support, and signs of barotrauma, for example, pneumothorax, pneumomediastinum, and subcutaneous emphysema. Eventually, she underwent surgery not only because her vital signs continued to worsen, but also to exclude diaphragmatic injuries due to ICD, hollow viscus perforation, and abdominal compartment syndrome [5].

## Conclusions

As we have shown, a case of tension pneumoperitoneum probably caused by HFOV was reported. Awareness of this condition should be included in the differential diagnosis. We suspected that the condition could occur in a patient supported with HFOV at a low pressure of mean alveolar pressure.

## Data Availability

The authors have no material or software used in this report.
